# Optimal design of on‐scalp electromagnetic sensor arrays for brain source localisation

**DOI:** 10.1002/hbm.25586

**Published:** 2021-07-10

**Authors:** Leandro Beltrachini, Nicolas von Ellenrieder, Roland Eichardt, Jens Haueisen

**Affiliations:** ^1^ Cardiff University Brain Research Imaging Centre (CUBRIC), School of Physics and Astronomy Cardiff University Cardiff; ^2^ Montreal Neurological Institute and Hospital McGill University Montreal Canada; ^3^ Institute of Biomedical Engineering and Informatics Ilmenau University of Technology Ilmenau Germany

**Keywords:** array processing, Cramér‐Rao bound, electroencephalography, magnetoencephalography, optically pumped magnetometers, source localisation

## Abstract

Optically pumped magnetometers (OPMs) are quickly widening the scopes of noninvasive neurophysiological imaging. The possibility of placing these magnetic field sensors on the scalp allows not only to acquire signals from people in movement, but also to reduce the distance between the sensors and the brain, with a consequent gain in the signal‐to‐noise ratio. These advantages make the technique particularly attractive to characterise sources of brain activity in demanding populations, such as children and patients with epilepsy. However, the technology is currently in an early stage, presenting new design challenges around the optimal sensor arrangement and their complementarity with other techniques as electroencephalography (EEG). In this article, we present an optimal array design strategy focussed on minimising the brain source localisation error. The methodology is based on the Cramér‐Rao bound, which provides lower error bounds on the estimation of source parameters regardless of the algorithm used. We utilise this framework to compare whole head OPM arrays with commercially available electro/magnetoencephalography (E/MEG) systems for localising brain signal generators. In addition, we study the complementarity between EEG and OPM‐based MEG, and design optimal whole head systems based on OPMs only and a combination of OPMs and EEG electrodes for characterising deep and superficial sources alike. Finally, we show the usefulness of the approach to find the nearly optimal sensor positions minimising the estimation error bound in a given cortical region when a limited number of OPMs are available. This is of special interest for maximising the performance of small scale systems to ad hoc neurophysiological experiments, a common situation arising in most OPM labs.

## Graphical abstract

We evaluated the advantages of hybrid OPM and EEG sensor arrays for source localisation using statistical bounds. Results show that EEG and OPMs are complementary, and that the CRB provides a theoretical framework for their flexible design.

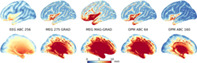

## 1 INTRODUCTION

Optically pumped magnetometers (OPMs) are revolutionising the way we measure magnetic brain signals. Since their introduction in the brain imaging field in 2018 (Boto et al., 2018), they have provided insights into cerebral activity difficultly achieved by the well‐established SQUID‐based magnetoencephalography (MEG) sensors. The reasons are simple: OPMs are portable and can be attached directly on the scalp. This allows (i) to reduce the distance between the sensors and the brain current generators by approximately 15 mm, with a consequent gain in signal‐to‐noise ratio (SNR) and (ii) the subject to move more freely during the acquisition, expanding the spectrum of MEG experiments to those previously achievable only by electroencephalography (EEG). Moreover, they do not require cryogenic cooling for functioning, making their running costs negligible. All these advantages are making OPM technology particularly attractive in a number of studies, from epilepsy (Vivekananda et al., 2020) to virtual reality (Roberts et al., 2019).

As is the case with any new technique, scientists have been interested in quantifying the benefits over its predecessor. With regard to OPM‐MEG, this means to compare their performance with conventional SQUID‐MEG for characterising sources of brain activity. This task is of particular importance at this early developmental stage for determining optimal array designs that allow minimal source reconstruction errors. Such a comparison has been assessed through a number of metrics, for example, the SNR (Hill et al., 2020), the free energy (Duque‐Munoz et al., 2019), the total information (TI) conveyed by the array (Iivanainen, Stenroos, & Parkkonen, 2017), and the spatial information density, a generalisation of the TI (Riaz, Pfeiffer, & Schneiderman, 2017). However, none of these works have studied how the OPM arrangement impacts directly on the variance of the reconstructed source considering arbitrary arrays and source estimation algorithms, which can consequently inform optimal sensor positioning systems.

In this article, we investigate the advantages of OPM‐MEG systems by means of the Cramér‐Rao bound (CRB). This metric provides a lower bound on the variance of the estimated source parameters achievable by any unbiased estimator, that is, regardless of the algorithm used (as long as it is unbiased). Furthermore, the CRB is an asymptotically tight bound, achievable by certain algorithms if the number of samples is large enough (Van Trees & Bell, 2013). These properties have made the CRB a standard tool in performance analysis and engineering design in a variety of fields, ranging from sonar to diffusion MRI (Alexander, 2008; Van Trees, 2002), and E/MEG in particular (Beltrachini, Von Ellenrieder, & Muravchik, 2011; Beltrachini, von Ellenrieder, & Muravchik, 2013; Dogandzic & Nehorai, 2004; Hochwald & Nehorai, 1997; Muravchik & Nehorai, 2001; von Ellenrieder, Muravchik, & Nehorai, 2005). Here, we computed the CRB to quantify the impact of instrument and modelling noise in the variance of the position of the localised source, and employed it in three relevant problems. First, to assess the performance of whole‐head standard OPM‐based MEG arrays based on existing on‐scalp sensor positioning setups. Although such large arrays are currently scarce (Hill et al., 2020), the CRB allows to quantify the improvements on source localisation compared with SQUID‐based montages resembling commercially available systems, as well as with high density EEG (hdEEG). Secondly, we exploited the complementarity found between EEG and OPM‐MEG to design optimal OPM and hybrid OPM/EEG arrays minimising the localisation error throughout the brain. This represents a major engineering problem in the area to which the CRB presents suitable and optimal solutions. Finally, we utilised the CRB for solving the more practical problem of finding optimal sensor positions when a specific brain region is targeted. We show that the CRB is a robust tool to design the optimal array for any given number of sensors and particular application, enhancing the flexibility of available equipment.

## 2 METHODS

### 2.1 The Cramér‐Rao bound

The main objective in electromagnetic brain mapping is to characterise sources of electrical activity based on a set of measurements and a head and sensor models. Under certain assumptions, such sources can be expressed mathematically by a function of parameters representing their position and strength. Unfortunately, the estimation of these parameters from real data, usually referred to as the inverse problem in E/MEG, does not have a unique solution. This has led the community to present a myriad of methods based on different hypotheses and simplifications, and consequently leading to different results (Grech et al., 2008). For this reason, a comprehensive analysis on the impact of sensor type and position on source estimates would demand an immense effort.

The CRB solves this problem by establishing the optimal performance achievable by any unbiased estimator regardless of the algorithm used (Van Trees & Bell, 2013). Although the CRB is not the tightest deterministic bound (a property attributable to the Barankin bound [Barankin, 1949]), it has been shown to provide tight results achievable by existing algorithms (e.g., Beltrachini et al., 2011; Beltrachini et al., 2013; Fernández‐Corazza, Beltrachini, von Ellenrieder, & Muravchik, 2013). Let $\vartheta \in {\mathbb{R}}^{N}$ be a vector with elements $\vartheta_{i}$, i=1,…,N, defining the source of brain activity (both intensity and location). As we aim to characterise the endogenous current source from a given set of measurements, we look to estimate ϑ. In this context, the CRB states that

(1) $$E\left\{\left(\vartheta -\;\hat{\vartheta }\right){\left(\vartheta -\;\hat{\vartheta }\;\right)}^{T}\right\}\geq \mathrm{CRB}\left(\vartheta \right),$$

where the inequality means that the difference between matrices on the left and right hand sides is positive semidefinite. In particular, the diagonal elements of $\mathrm{CRB}\left(\;\vartheta \;\right)$ establish the minimum variance on each source parameter $\vartheta_{i}$.

The calculation of the CRB depends on a number of ingredients, including the source and signal models, the sensor type and positions, and the numerical methodology employed to discretise the E/MEG governing equations (also known as E/MEG forward problem). Here, we based our analysis on Muravchik and Nehorai's study (Muravchik & Nehorai, 2001), which assumed unconstrained dipolar sources of electrical activity and electromagnetic signals corrupted by zero‐mean Gaussian noise with standard deviation $\sigma_{b}$ and $\sigma_{e}$ for MEG and EEG, respectively, representing instrument noise. By unconstrained sources we mean current generators whose locations are not assumed restricted to a surface. In this case, the parameter vector describing the source contains six elements, the first three defining the source position, and rest describing its moment. As for the numerical solution of the forward problem, we used the boundary element method (BEM). We direct the interested reader to (Muravchik & Nehorai, 2001) for further details.

Once the CRB is computed for any given current source, we can use it to calculate meaningful error metrics related to the minimum variance achievable of the estimated position. In particular, we can obtain the volume of the 95% probability concentration ellipsoid, defined as the volume of the ellipsoid that encloses the mean of an unbiased estimate with 95% probability, and given by

(2) $$V_{95}=\frac{4}{3}\pi c^{3}{\left|R\right|}^{1/2},$$

where c=2.8 and R is the 3×3 submatrix of the CRB corresponding to the source location variables (Muravchik & Nehorai, 2001; Van Trees & Bell, 2013). For simplicity, we define the *equivalent uncertainty radius*, $r_{95}$, as the radius of the sphere with volume equal to $V_{95}$,

(3) $$r_{95}=c{\left|R\right|}^{1/6}.$$

Since $r_{95}$ defines the minimum volume containing the estimated source with 95% probability, we aim it to be as small as possible. In other words, the smaller $r_{95}$ is, the better the source location estimate can be.

### 2.2 Head model and discretisation

We generated a head model based on the Colin27 high resolution MRI segmentation of the Montreal Neurological Institute (Aubert‐Broche, Evans, & Collins, 2006). Images were employed to obtain three layers representing the scalp, skull, and brain. The innermost layer was tessellated in 40,960 triangular elements to avoid numerical errors for eccentric sources (Haueisen et al., 1997), whereas the other two consisted on 5,120 triangles. In the case of MEG, we employed the single shell model (Hämäläinen & Sarvas, 1989) and used linear elements (Ferguson, Zhang, & Stroink, 1994). In the case of EEG, BEM matrices were computed using the three layered model, linear elements (de Munck & Peters, 1993), and the isolated skull approach (Meijs et al., 1989). The adopted electrical conductivities were 0.011 S/m for the skull and 0.33 S/m for the scalp and brain (McCann, Pisano, & Beltrachini, 2019). Thirty‐three thousand dipolar sources were uniformly located on each lobe's pial surface as obtained with FreeSurfer (Dale, Fischl, & Sereno, 1999). It is worth noting that sources were assumed unconstrained to any surface for their estimation, and their localisation on the pial surface was done just for clarity in the presentation of results.

### 2.3 Sensor arrays

**FIGURE 1:**
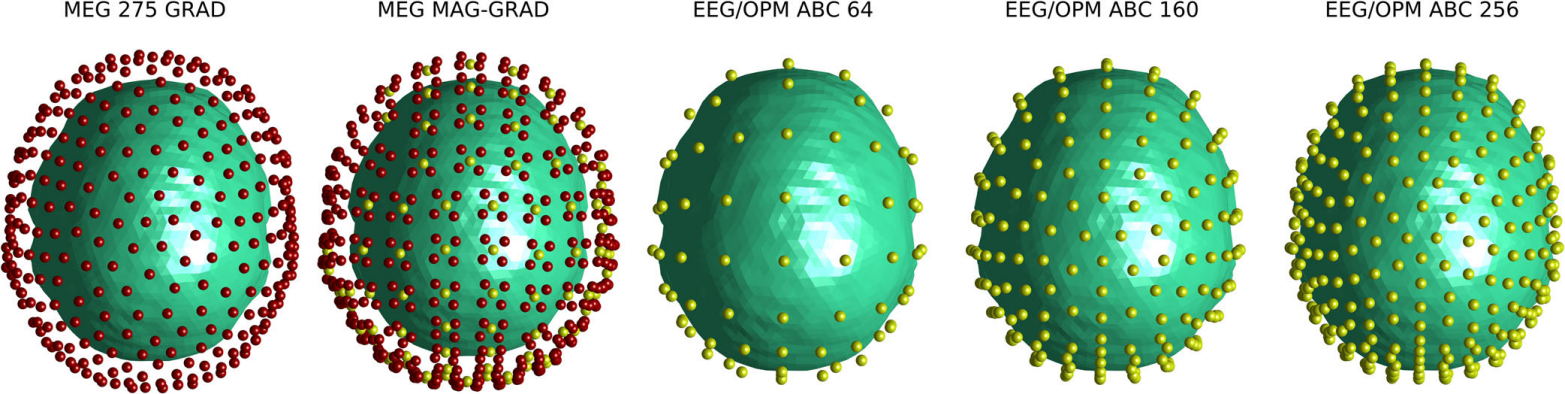
Sensor setups employed in the study. From left to right: 275 axial gradiometers (only coils closest to scalp are shown), 102 axial magnetometers and 204 planar gradiometers, ABC 64, 160 and 256 montages. Red dots indicate gradiometer coils, whereas yellow dots represent magnetometers or EEG electrodes (whenever corresponds)

We considered five standard acquisition setups, all shown in Figure 1. In the case of SQUID‐based sensors, we utilised two commonly found systems. The first, hereafter referred to as MEG 275 GRAD, consisted of 275 first‐order axial gradiometers with 53 mm baseline (i.e., distance between coils), with the closest coils located at 23 mm from the scalp. Sensors in this model were arranged as in the 275 channel CTF MEG system (Vancouver, Canada). The second model, named MEG MAG‐GRAD, was composed of 102 magnetometers measuring the axial component of the magnetic field and 204 planar gradiometers with 1.7 cm baseline, all located at 23 mm from the scalp and arranged as in the MEGIN MEG system (MEGIN Oy, Helsinki, Finland). As for the OPM/EEG sensor locations, we chose the positioning systems comprising 32 (not shown), 64, 160, and 256 sensor locations arranged as in the corresponding BioSemi caps, known as ABC layouts (BioSemi B.V., Amsterdam, Netherlands). The ABC 64 and 160 montages allowed to study the performance of medium and high density whole‐head arrays that may be financially and ergonomically feasible with the OPM technology as it stands (the minimum distance between sensors was larger than 1.6 cm for ABC 160 in the Colin27 model). The ABC 160 system was also used to generate a discrete set of predefined locations where to place the on‐scalp magnetometers to maximise the sensitivity to a particular brain region, as well as a basis for the hybrid OPM/EEG array (see Section 2.4). OPMs were modelled as magnetometers measuring the magnetic field in the axial and one tangential direction (randomly chosen in this study; see Section 4 and Figure S1) and located at a distance of 6 mm from the scalp, emulating the second generation sensors provided by QuSpin Inc. (Colorado). The ABC 256 system was used to calculate the CRB for hdEEG, which has been shown to provide results competitive with MEG (e.g., Hedrich et al., 2017). Sensors were located utilising fiducial markers and employing instructions available in the vendor's website (as in the case of the ABC layouts). Point sensor and electrode models were used.

### 2.4 Experiments

We conducted three experiments based on the CRB framework. Firstly, we used it to compare the performance of whole‐head OPM‐based MEG systems with existing SQUID‐MEG and hdEEG arrays. This was done by calculating the equivalent uncertainty radius for sources located on the cortex and the arrays introduced in Figure 1. Such a comparison is important for quantifying the pros and cons of OPMs over established systems from a source localisation perspective, presenting the basis for engineering optimal prototypes.

In the second experiment, we employed the CRB to design optimal whole‐head sensor arrangements using OPMs and a combination of OPMs and EEG electrodes. Sensor positions were constrained to the ABC 160 standard (with a maximum of 160 sensor positions), and on‐scalp magnetometers were sequentially included such that each new addition minimised the median of $r_{95}$ throughout the entire cerebral cortex the most. In other words, at each iteration, the median was calculated for all possible remaining sensor locations and the one that minimised the median the most was then chosen and fixed, before the next iteration of the algorithm started. In the case of the hybrid OPM/EEG array, we considered the remaining sensor positions (i.e., those without an OPM) as EEG electrodes, totalling 160 E/MEG measurements. We computed the error as a function of the number of sensors and compared it with the corresponding error for the aforementioned SQUID‐based and hdEEG arrays.

Finally, in the third experiment, we found the optimal arrangement for an arbitrary number of sensors minimising the lower bound on the source localisation variance for brain current generators in specific cortical regions. This is of particular interest for most MEG scientists working in OPM technology that do not count with enough sensors for building a whole‐head system, and therefore in need of ad hoc sensor positioning procedures depending on the study. To do so, we employed Destrieux's cortical surface parcellation (Destrieux, Fischl, Dale, & Halgren, 2010) as provided by FreeSurfer and computed the median of the equivalent uncertainty radius for sources in the selected regions. As before, sensor positions were constrained to the ABC 160 standard, and OPMs were sequentially included such that each new addition minimised the CRB metric the most. To illustrate the applicability of the approach, we considered two different scenarios focused on the primary motor cortex (label 29 in Destrieux's atlas) and on basal temporal structures (labels 21, 22, 23, 37, 43, 50, 51, 60, 61, and 72 in Destrieux's atlas).

In all experiments, we adopted the MEG noise standard deviation, $\sigma_{b}$, equal to $6\;\text{fT}/\sqrt{\mathrm{Hz}}$ and $4\;\text{fT}/\sqrt{\mathrm{Hz}}$ for OPMs and SQUID measurements, respectively (Shah & Wakai, 2013; Vrba & Robinson, 2002), the EEG noise standard deviation $\sigma_{e}=50\;\mathrm{nV}/\sqrt{\mathrm{Hz}}$ (Niedermeyer & Da Silva, 2004; Pourahmad & Mahnam, 2016), and bandwidth BW=40Hz (Vrba & Robinson, 2002).

## 3 RESULTS

### 3.1 Comparison between standard whole‐head arrays

**FIGURE 2:**
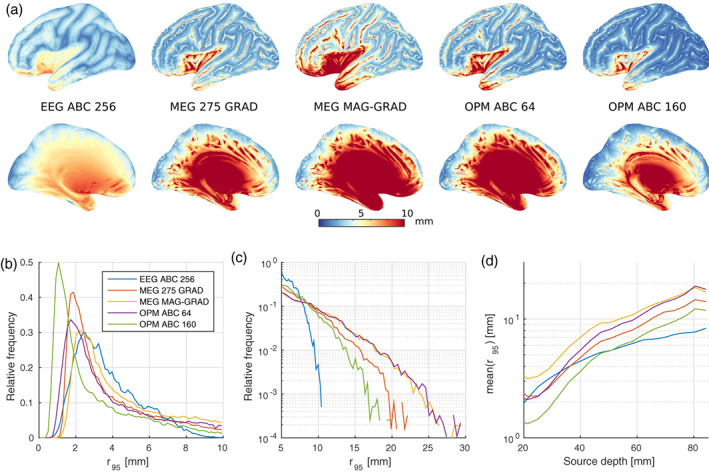
(a) Equivalent uncertainty radius for sources located on the left hemisphere and the following arrays (from left to right): EEG ABC 256 (hdEEG), MEG 275 GRAD, MEG MAG‐GRAD, OPM ABC 64 and OPM ABC 160. (b,c) Normalised histogram of the equivalent uncertainty radius for all sources in linear (b) and semi‐logarithmic (c) scales. (d) Mean value of $r_{95}$ as a function of the source depth (binned every 5 mm)

Figure 2a shows the equivalent uncertainty radius for sources located on the left hemisphere and employing the whole‐head acquisition systems of Figure 1. The differences amongst sensor arrays and, most importantly, sensor types, are remarkable. On the one hand, hdEEG presented a smooth (and relatively low) equivalent uncertainty radius for eccentric sources, which naturally increased for deeper structures. On the other hand, all MEG arrays (regardless of the sensor type) showed, compared to hdEEG, better performance for eccentric and tangentially oriented sources, but worst performance for radially oriented sources (to which MEG is known for having lower sensitivity to) and generators located in deep brain regions.

In Figure 2b–d, we display a set of metrics that provide a quantitative means for comparing the performance of such acquisition systems. Figure 2b,c shows the normalised histograms of the equivalent uncertainty radius in linear and semi‐logarithmic scales, respectively, whereas Figure 2c depicts the mean value of $r_{95}$ as a function of the source depth (defined as the minimum distance between the source and the scalp) binned every 5 mm. From Figure 2b, it is clear that all MEG systems presented lower modes (i.e., values of $r_{95}$ corresponding to the histogram's peak) than hdEEG. In particular, the OPM ABC 160 array showed the lowest mode, while the OPM ABC 64 exhibited results comparable to the commercial SQUID‐based systems. However, it can also be seen that the relative frequency of $r_{95}$ decays faster for hdEEG than for all MEG arrangements. This can be appreciated in more detail in Figure 2c, where hdEEG resulted in a maximum equivalent uncertainty radius of approximately 11 mm, increasing to approximately 18 mm for the best performing MEG system (OPM ABC 160). This difference highlights the complementarity between EEG and MEG techniques for characterising current generators across the entire brain. Figure 2d stresses this concept even more by contrasting the benefits of each imaging modality and array as a function of the source depth.

### 3.2 Design of optimal whole‐head systems

**FIGURE 3:**
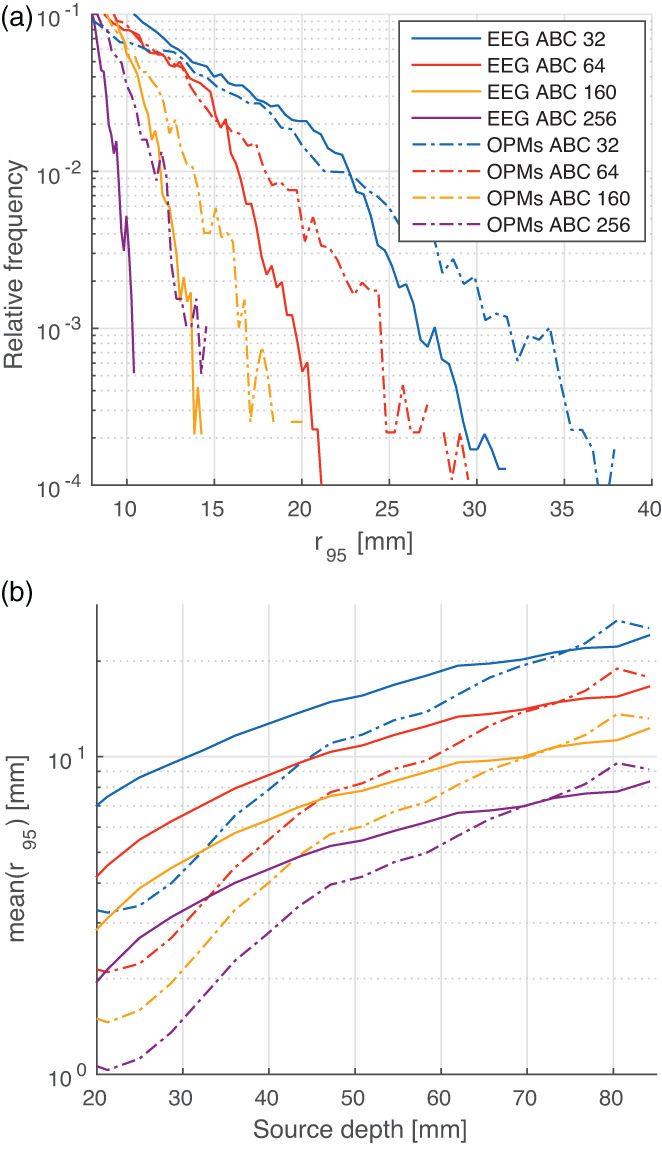
(a) Normalised histogram of the equivalent uncertainty radius for all sources in semi‐logarithmic scale. (b) Mean value of $r_{95}$ as a function of the source depth (binned every 5 mm)

Based on the previous results, we further evaluated the complementarity between EEG and OPM systems for source localisation considering the same electrodes/sensors layouts. Figure 3a,b displays the normalised histograms of the equivalent uncertainty radius in semi‐logarithmic scale and the mean value of $r_{95}$ as a function of the source depth, respectively. While OPMs presented clear advantages over EEG electrodes for most source locations, EEG still provided benefits for deep sources, outperforming MEG for current generators at a distance larger than 70 mm from the scalp. However, the most noticeable aspect of Figure 3b is that EEG generates complementary information to OPM‐based arrays for source localisation provided that the number of electrodes is equal or larger than the number of on‐scalp magnetometers, as seen from the direct comparison of curves for different arrays (e.g., EEG ABC 160 has a lower mean value of $r_{95}$ than OPMs ABC 160, 64, and 32 for sources deeper than 72, 46, and 32 mm, respectively).

**FIGURE 4:**
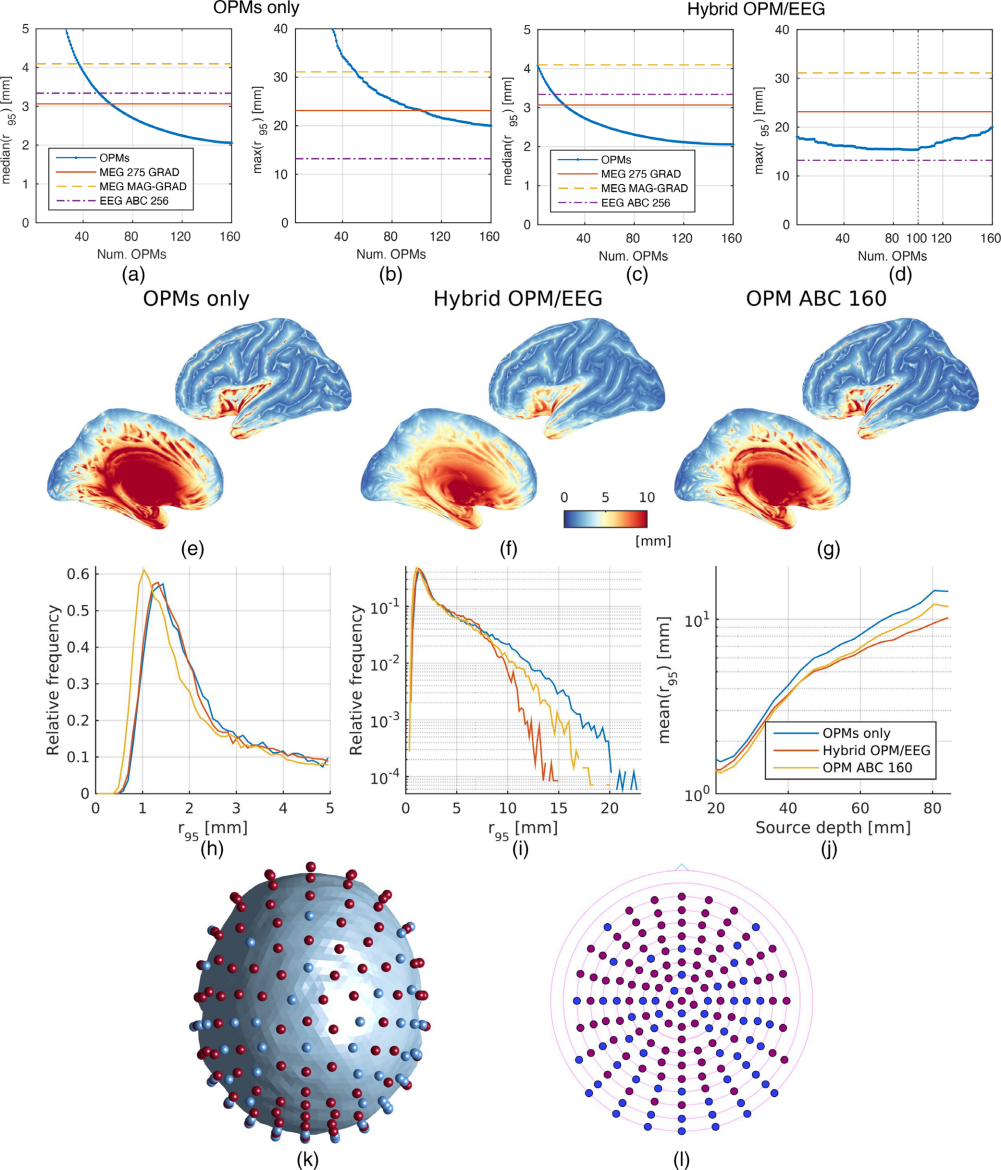
Whole‐head OPM and hybrid OPM/EEG designs. (a–d) Error metrics for the OPM and hybrid OPM/EEG systems as a function of the number of on‐scalp magnetometers considered. The median and maximum of $r_{95}$ are presented for both systems, together with the metrics corresponding to commercially available arrays (with different colours), which are constant and independent on the number of OPMs. (e–g) Spatial distribution of the equivalent uncertainty radius for the OPMs only (e), hybrid OPM/EEG (f), and full OPM ABC 160 (g) arrays, the first two employing 100 on‐scalp magnetometers. (h–i) Normalised histogram of the equivalent uncertainty radius for all sources in linear (h) and semi‐logarithmic (i) scales. (j) Mean value of $r_{95}$ for the three systems as a function of the source depth (binned every 5 mm). (k–l) Optimal hybrid OPM/EEG array sensor positions (k) and layout (l). EEG electrodes and OPMs are represented with blue and red dots, respectively

Figure 4a–d present the median and maximum of the equivalent uncertainty radius based on OPM (a,b) and hybrid OPM/EEG (c,d) systems. It can be seen that the incorporation of EEG electrodes to the OPM setup benefits the overall performance not only by reducing the median of $r_{95}$, but also its maximum. Unsurprisingly, the more OPMs were included, the lower the median of $r_{95}$ resulted. However, the maximum of the equivalent uncertainty radius does present an optimal value for the hybrid system, which consisted of 100 on scalp magnetometers for the layout employed (Figure 4d). The performance of the resulting optimal systems for 100 OPMs are shown in Figure 4e–j. The spatial distribution maps of $r_{95}$ (Figure 4e–g) reveal that the hybrid option is not only competitive to the OPM ABC 160 array, but also presented less error for radially oriented generators and deep sources. The optimal hybrid OPM/EEG array for 100 OPMs is shown in Figure 4k–l. It is apparent that the algorithm favours the position of OPMs in scalp regions closer to the cortex (i.e., on top, frontal, and lateral scalp regions), whereas EEG electrodes are preferred in areas with better access to medial and temporal structures, such as on the back side of the head.

### 3.3 Optimal ad hoc arrays

**FIGURE 5:**
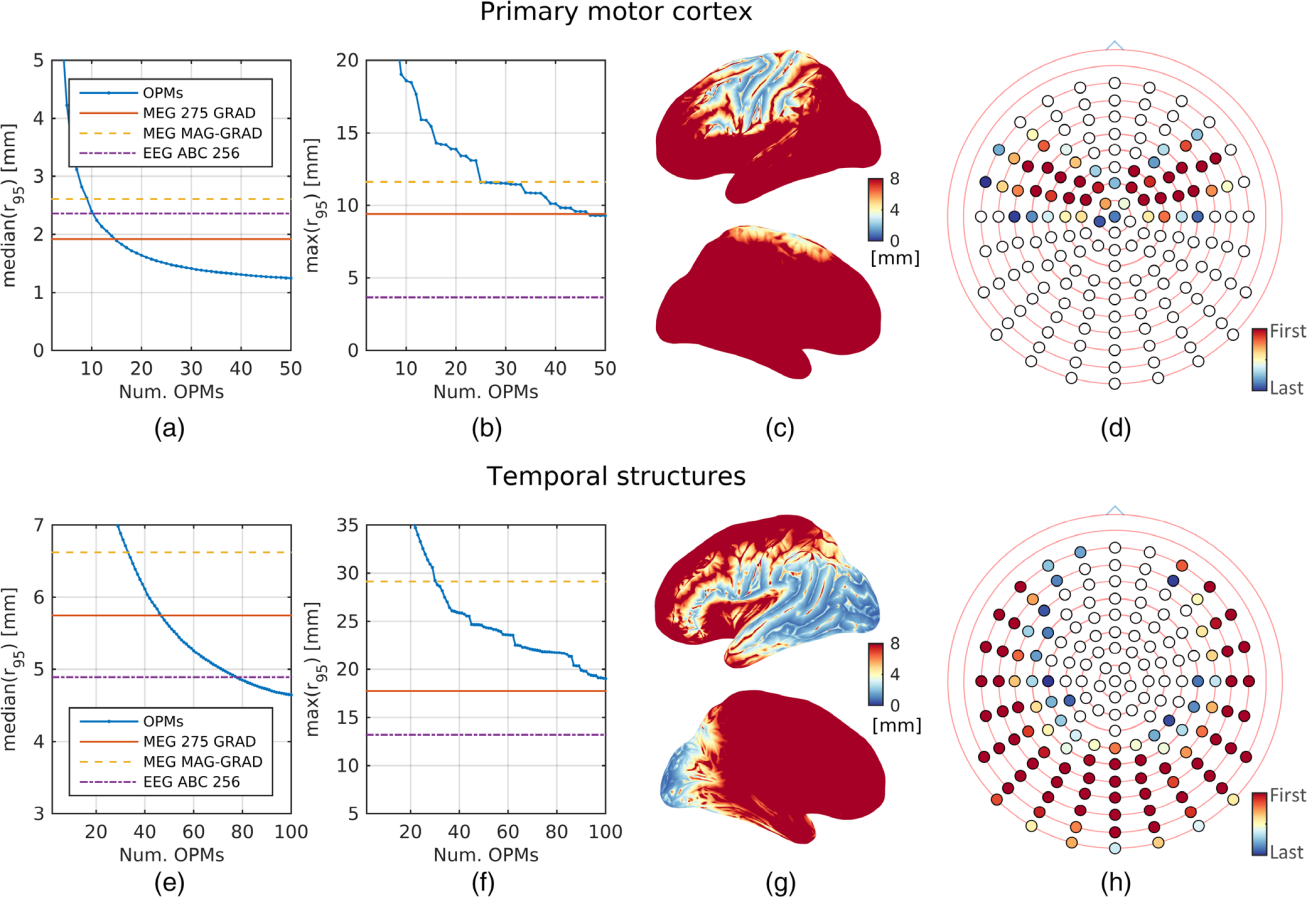
Performance of OPM MEG systems for estimating sources in the (a,b) primary motor cortex and (e–f) basal temporal structures. (a,b,e,f) Variation of the median (a,e) and maximum (b,f) of $r_{95}$ as a function of the number of optimally placed OPMs. Error metrics for the EEG ABC 256, MEG 275 GRAD, and MEG MAG‐GRAD arrays, which are constant and independent on the number of OPMs, are also presented. (c,g) Spatial representation of $r_{95}$ for the system comprising as many OPMs as needed for obtaining similar median of the equivalent uncertainty radius as the MEG MAG‐GRAD array. Only the left hemisphere is shown from both exterior and interior viewpoints. (d,h) Location of the optimally selected OPMs overlaid on the schematic ABC 160 sensor configuration. Colours correspond to the order on which they were included (same as in a,e), with dark red indicating the sensors needed to achieve a similar value of the median of $r_{95}$ as the MEG 275 GRAD system

Results to the third experiment are presented in Figure 5 for both primary motor cortex and basal temporal structures. The variation of the median (Figure 5a,e) and maximum (Figure 5b,f) of the equivalent uncertainty radius is plotted as a function of the number of OPMs for both regions of interest, together with the metrics corresponding to the commercially available arrays (with different colours). Based on the median, we can conclude that at least 15 and 47 on‐scalp magnetometers would be required to reach a similar performance to the best performing commercial MEG system (MEG 275 GRAD) for studying the primary motor cortex and basal temporal structures, respectively. However, this does not represent a comprehensive comparison, as evidenced from the number of OPMs needed to secure a maximum of $r_{95}$ comparable to MEG 275 GRAD. In Figure 5c,g, we present the equivalent uncertainty radius obtained for OPM systems comprising as many sensors as needed for reaching a similar median of $r_{95}$ as the MEG MAG‐GRAD array. Only the left hemisphere is shown from both exterior and interior viewpoints (symmetric results are obtained for the right hemisphere). The corresponding locations of the sensors sequentially included in the system (and minimising the error metric in the cortical areas) are shown in Figure 5d,h, with red dots indicating the sensors needed to achieve a similar median as that of MEG 275 GRAD. It can be seen that both number and location of sensors, as well as the region of interest, are important for optimising the experimental setup.

## 4 DISCUSSION

Performance bounds are integral parts of array signal processing theory and practice, allowing quantitative comparisons between engineering designs in terms of the optimal capability achievable. Here, we employed its most famous exponent, the CRB, to evaluate and design E/MEG arrays minimising the source localisation errors. The main advantage of this approach lies in its capability to provide optimal performance metrics on the solution of the electromagnetic source localisation problem (also known as E/MEG inverse problem, E/MEG‐IP) based on the sensor type, number, and arrangement, but independently of the algorithm employed. This is of remarkable value in a field in which new E/MEG‐IP techniques arise regularly, generating results valid for any unbiased methodology. The CRB framework was used to compare the capabilities of OPM arrays with others generally utilised in the field, such as hdEEG and SQUID‐based systems, as well as to generate optimal sensor arrangements suited to different needs.

In the particular case of whole‐head arrangements, we found that OPM arrays based on standard EEG layouts produced noticeable outcomes. More explicitly, we obtained that using 64 sensors placed according to the ABC standard resulted in better source estimates than MEG MAG‐GRAD for most eccentric sources, and comparable to MEG 275 GRAD. The situation changed for sources at a distance equal or larger than 30 mm from the scalp, where MEG 275 GRAD outperformed OPM ABC 64. The reason originates in the number of sensors available in the commercial system, which allows a better coverage of the cerebral cortex. This problem is clearly solved by the OPM ABC 160 montage, which produced outstanding estimates with almost half the number of magnetometers found in commercial arrays. Although hypothetical, the OPM ABC 160 concept is not difficult to envisage, with arrays composed of almost 50 sensors being constructed only 2 years after their introduction in the field (Hill et al., 2020).

It has been known for long time that EEG and MEG are not competing but complementary imaging techniques due to their dissimilar spatial sensitivities (e.g., Hari & Puce, 2017; Hunold et al., 2016). However, the advent of OPM technology allowed to imagine that on‐scalp MEG may make the use of EEG redundant, providing insights into deep current generators as never before. Results from the first experiment showed that such a hypothesis is still false for the current technology, with hdEEG presenting benefits over high density OPM arrays for both deep and radially oriented sources. This can be noted from the smoother estimation bounds in Figures 2a,d, as well as in the lower value of the equivalent uncertainty radius' maximum in Figure 2c. In the case of eccentric sources, the EEG ABC 256 array presented an equivalent uncertainty radius smaller 5 mm regardless of their orientation (seen in blue and white colours). This was not the case for any of the MEG scanners tested, all exhibiting unacceptably larger bounds for all radial generators (seen as white and red lines in Figure 2a). Moreover, hdEEG provided noteworthy results for deep sources, outperforming even OPM ABC 160 for regions at a distance equal or larger than 50 mm from the scalp. Similar conclusions can be extracted from Figure 3, which highlights the advantages of incorporating EEG to OPM systems whenever the number of EEG electrodes is larger or equal than the number of OPMs. While on‐scalp magnetometers allow to reduce the mode of $r_{95}$, EEG sensors are required to minimise its maximum, with implications to both deep and eccentric brain regions.

Results from the first experiment allowed to realise the importance of integrating EEG and OPMs for generating optimal portable whole‐head systems. This led us to design two nearly optimal on‐scalp electromagnetic arrays sensitive to signals generated throughout the brain: one built from OPMs (as it is currently the gold standard [Hill et al., 2020]) and another complemented with EEG electrodes. As expected, the more OPM sensors used, the better results the OPM array achieved. However, this was not the case for the hybrid system, for which we found a compromise between the number of magnetometers and electrodes for reaching the minimum of both mode and maximum of the equivalent uncertainty radius. This optimal relation is clearly dependent on the layout utilised, and resulted in 100 OPMs and 60 EEG electrodes for the ABC 160 setup. The results shown in Figure 4e–j confirm the finding, with the hybrid OPM/EEG array presenting improvements over the OPM and, more importantly, the OPM ABC 160 arrays. The incorporation of EEG electrodes reduced the source estimation error for both deep and radially oriented current sources while maintaining the performance for other generators. This is a major engineering finding that will certainly guide the construction of fully integrate prototypes incurring at not much extra cost than OPM systems.

In addition to comparing whole‐head sensor arrangements, we utilised the CRB to solve the more pragmatic problem of finding the optimal sensor positions for minimising the source localisation error when a limited number of OPMs are available. This situation is especially relevant to research groups experimenting with the technology interested in localising sources due to tasks targeting specific brain regions. We employed the CRB to find a nearly optimal set of sensor positions minimising the E/MEG‐IP error bound within the discrete set of possible locations provided by the ABC 160 layout. Results indicated that the number and place of such sensors depend on the cortical region of interest, requiring less for eccentric sources and more for deep generators. In the special case of the primary motor cortex, only 15 OPMs were found necessary to reduce the median of the equivalent uncertainty radius to the level of the best SQUID‐based equipment. The number increased to 47 in the case of focusing on basal temporal structures. However, as pointed out by the previous experiments, the median is not sufficient to characterise the error bound distribution, with the maximum also being of great importance. The latter factor was found much more difficult to reduce to the level of the commercial array, requiring 47 and more than 100 to reach the same value as the MEG 275 GRAD system for the motor and temporal regions, respectively. Then, although OPM arrays can generate results comparable to SQUID MEG with only a limited number of sensors, one must remain cautious regarding the actual limitations of ad hoc systems. Even so, these findings are expected to impact on a number of studies centred on particular brain regions, such as brain computer interface (Clerc, Bougrain, & Lotte, 2016), focal cortical dysplasia (Vivekananda et al., 2020), and emotion processing (Ibáñez et al., 2011).

The utilisation of EEG positioning setups to constrain the location of the OPMs presents a number of benefits, such as the use of a common naming reference that aid in the dissemination and reproducibility of the results, as well as the consideration of a discrete and low dimensional space where to look for the optimal solution. Moreover, since all sensors need a corresponding holder, this option seems more practical than assuming free sensor positioning. Here, we used the CRB to find nearly optimal sensor locations within the ABC 160 layout for sources placed in cortical regions by sequentially adding the OPM that minimised the overall metric. Consequently, the resulting positions are not optimal by definition, since the algorithm assumes that any sensor arrangement will be also selected for any larger optimal configuration. Nevertheless, this resulted in a quicker implementation, which is needed for building an accessible tool with manageable computing requirements. Even more, this approach seems better suited for practical applications in which the number of sensors available at any moment may vary due to technological glitches, as we have personally experienced with state‐of‐the‐art OPMs. The use of a more thorough methodology, such as combinatorial multilevel optimisation (Eichardt et al., 2019), is planned for future development.

Additionally, the use of EEG montages as the base setup for arbitrary OPM systems raises controversies around their practicality and feasibility. Considering OPMs with 13 × 17 mm^2^ contact base (as the zero field magnetometers commercialised by QuSpin) plus 2 mm on each side accounting for the corresponding holder, there is space for approximately 335 sensors to be placed on an adult's head (with a total area of approximately 0.096 m^2^ based on the head model utilised). Although this indicates that layouts as dense as the ABC 256 are achievable, it may represent a very challenging engineering task to make them a reality. This led us to use the ABC 160 system, whose inter electrode/sensor distance allows for a full OPM array. Additional computations (Figure S1) have shown that the effect of rotating the tangential axis is negligible for whole‐head systems, in line with other studies (Eichardt et al., 2019). This flexibility in the sensor orientation facilitates the assemble of high density OPM arrays, and therefore worth exploiting. Besides, the supplementary figure highlights the theoretical advantages of measuring the complete vector field at each sensor, which is now being explored in some research groups (Labyt et al., 2019). Notwithstanding, it is important to acknowledge constraints other than the space availability and sensor closeness, such as those related to sensor heating, weight, and crosstalk. In this regard, equidistant sensor systems used for dry EEG may present a valuable alternative (Fiedler et al., 2015).

It is worth noting that the CRB framework employed here does not come without limitations. First, as already discussed, the signal model assumed uncorrelated Gaussian noise representing mostly instrument and (up to some degree) geometrical modelling perturbations. Therefore, the methodology did not incorporate variability in the estimated parameters due to other error sources such as background activity (Beltrachini et al., 2013; de Munck, Vijn, & Lopes da Silva, 1992), sensor mislocation (Beltrachini et al., 2011) and tilting (Hill et al., 2020). All these perturbations are likely to increase the CRB metrics and consequently worth exploring in more detail. What is more, although this noise model is widely adopted and accepted, it is an idealised version of the real noise found in OPMs (and sensors in general), and therefore more work is needed to represent noise depending on the sensor technology (Eichardt et al., 2019; Oelsner et al., 2019). Second, we utilised the point electrode and sensor models, which are generally adopted in E/MEG, and in OPM research in particular (Duque‐Munoz et al., 2019; Hill et al., 2020; Pfeiffer et al., 2018). Even though the inclusion of the sensor geometry may have led to slightly more precise results, their impact is expected to be insignificant compared with other modelling approximations, such as the electrical conductivity values used (McCann et al., 2019) and the omission of the CSF compartment (Piastra et al., 2021). Third, we modelled sources of brain activity as unconstrained current dipoles. The use of such a simple model is required in the CRB context to avoid complicated expressions that may result difficult (or even impossible) to derive analytically. Consequently, this analysis gives an idea of the maximum sensitivity that can be expected from OPM and hybrid arrays for a single source. The separability and effect of cross‐talk of multiple sources, which could impede to achieve the sensitivity bound employed here, is not addressed by the presented methodology. Further work should focus on analysing this particular aspect of the arrays obtained, which could be noticeable on practical designs. Several source models were introduced to describe source generators more realistically, as those based on the multipolar expansion (Beltrachini, 2019; Jerbi, Mosher, Baillet, & Leahy, 2002). Nevertheless, dipolar current generators are generally the first choice for brain source characterisation, most prominently in epilepsy (Rampp et al., 2019; Vivekananda et al., 2020), as well as the basis for most E/MEG‐IP algorithms. Lastly, the algorithm for constructing the hybrid array may be optimised in several ways. One option is to incorporate variable weighting for the MEG signals depending on the source direction, resulting minimal for radial and deep sources, and maximal for tangential. This may lead to a more distributed pattern between electrodes and sensors. Another is to incorporate both EEG and MEG simultaneously in the optimisation, differently to the work here presented where OPMs were prioritised. Further work will analyse the impact that these changes may have in the resulting arrays.

## CONFLICT OF INTEREST

The authors declare no conflicts of interest.

## Supporting information

**FIGURE S1** Normalised histogram of the equivalent uncertainty radius for all sources. Results are presented for OPM ABC 64 and OPM ABC 160 arrays with different line styles, and considering measurements from both tangential directions of the field separately (*t*_1_ and *t*_2_) and at the same time (*t*_1_ and *t*_2_) (in addition to the axial component)

## Data Availability

Data and software used in this work has been properly cited within the manuscript.
