# Supplementary material for: Optimal design of on‐scalp electromagnetic sensor arrays for brain source localisation
Source: Hum Brain Mapp. 2021 Jul 10;42(15):4869–79. doi: 10.1002/hbm.25586 (PMC8449117; doi:10.1002/hbm.25586)
Supplement: Supplementary file 1 — FIGURE S1 Normalised histogram of the equivalent uncertainty radius for all sources. Results are presented for OPM ABC 64 and OPM ABC 160 arrays with different line styles, and considering measurements from both tangential directions of the field separately (t 1 and t 2) and at the same time (t 1 and t 2) (in addition to the axial component) [file HBM-42-4869-s001.pdf]

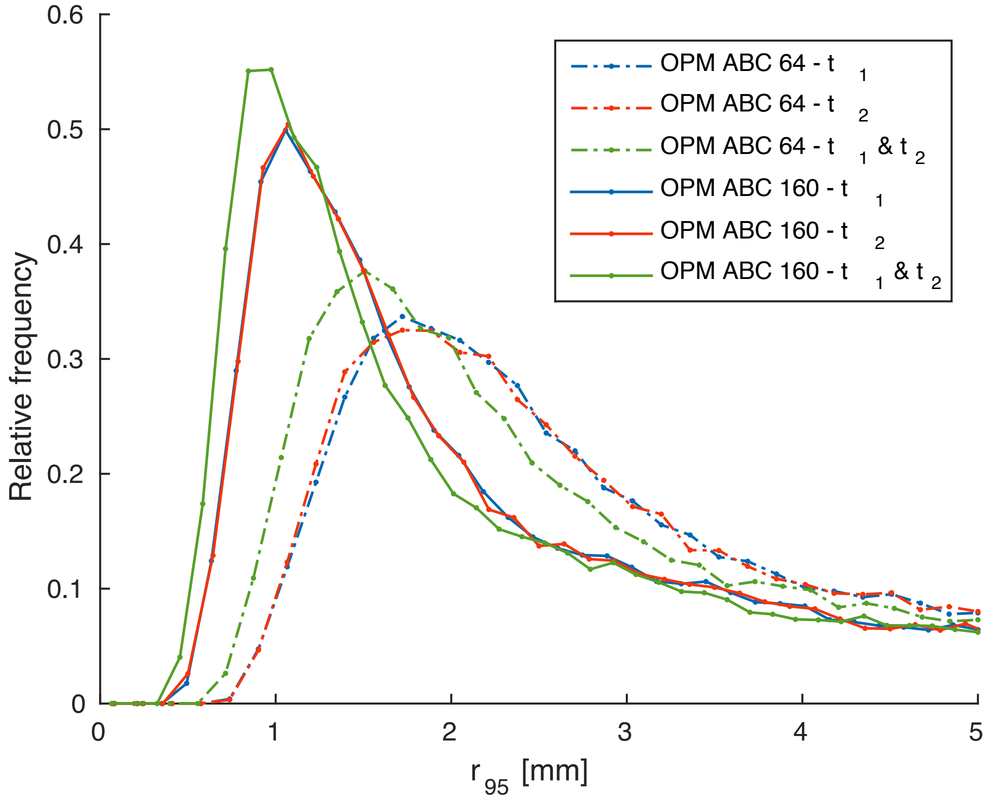

Supplementary figure. Normalised histogram of the equivalent uncertainty radius for all sources. Results are presented for OPM ABC 64 and OPM ABC 160 arrays with different line styles, and considering measurements from both tangential directions of the field separately (t_1 and t_2) and at the same time (t_1 & t_2) (in addition to the axial component).
